# Q waves are the strongest electrocardiographic variable associated with primary prophylactic implantable cardioverter-defibrillator benefit: a prospective multicentre study

**DOI:** 10.1093/europace/euab260

**Published:** 2021-11-29

**Authors:** Ari Pelli, M Juhani Junttila, Tuomas V Kenttä, Simon Schlögl, Markus Zabel, Marek Malik, Tobias Reichlin, Rik Willems, Marc A Vos, Markus Harden, Tim Friede, Christian Sticherling, Heikki V Huikuri, the EU-CERT-ICD Study Investigators, Elena Arbelo, Axel Bauer, Frieder Braunschweig, Josep Brugada, David Conen, Iwona Cygankiewicz, Michael Dommasch, Christian Eick, Panagiota Flevari, Tim Friede, Jan Galuszka, Jim Hansen, Robert Hatala, Markus Harden, Katerina Hnatkova, Heikki V Huikuri, Juhani M Junttila, Stefan Kääb, Gabriela Kaliska, Jaroslaw D Kasprzak, Andreas Katsimardos, Milan Kozak, Tomasz Kuczejko, Andrzej Lubinski, Jozef Martinek, Béla Merkely, Tomáš Novotný, Marek Malik, Peter Perge, Burkert Pieske, Pyotr Platonov, Pawel Ptaczyński, Dariusz Qavoq, L Rotkvić, Zoltan Sallo, Simon Schlögl, Georg Schmidt, Moritz Sinner, Rajeeva Sritharan, Stefan Stefanow, Christian Sticherling, Jesper Hastrup Svendsen, Martin Svetlosak, Janko Szavits-Nossan, Milos Taborsky, Anton Tuinenburg, Bert Vandenberk, Marc A Vos, Rik Willems, Stefan N Willich, Christian Wolpert, Markus Zabel, Ante Anic, Zoran Bakotic, Steffen Behrens, Dieter Bimmel, Sandro Brusich, Rüdiger Dissmann, Gerian Grönefeld, Przemyzlav Guzik, Svetoslav Iovev, Zrinka Jurisic, Thomas Klingenheben, Nikola Pavlović, Joachim Seegers, Robert H G Schwinger, Tchavdar Shalganov, Vassil Traykov, Vasil Velchev

**Affiliations:** 1 Research Unit of Internal Medicine, Medical Research Center Oulu, Oulu University Hospital and University of Oulu, PO Box 5000, FIN-90014 Oulu, Finland; 2 Biocenter Oulu, University of Oulu, Oulu, Finland; 3 Division of Cardiology, University Medical Center Göttingen Heart Center, Göttingen, Germany; 4 DZHK (German Center for Cardiovascular Research), partner site Göttingen, Göttingen, Germany; 5 National Heart and Lung Institute, Imperial College, London, UK; 6 Department of Internal Medicine and Cardiology, Masaryk University, Brno, Czech Republic; 7 Division of Cardiology, University Hospital Basel, Basel, Switzerland; 8 Department of Cardiovascular Sciences, University of Leuven and University Hospitals Leuven, Leuven, Belgium; 9 Medical Physiology, University Medical Center Utrecht, Utrecht, Netherlands; 10 Department of Medical Statistics, University Medical Center Göttingen, Göttingen, Germany

**Keywords:** Implantable cardioverter-defibrillator, Primary prevention, Mortality, Appropriate shock, Benefit, Heart failure, Electrocardiogram, Q wave, QT interval

## Abstract

**Aim:**

The association of standard 12-lead electrocardiogram (ECG) markers with benefits of the primary prophylactic implantable cardioverter-defibrillator (ICD) has not been determined in the contemporary era. We analysed traditional and novel ECG variables in a large prospective, controlled primary prophylactic ICD population to assess the predictive value of ECG in terms of ICD benefit.

**Methods and results:**

Electrocardiograms from 1477 ICD patients and 700 control patients (EU-CERT-ICD; non-randomized, controlled, prospective multicentre study; ClinicalTrials.gov Identifier: NCT02064192), who met ICD implantation criteria but did not receive the device, were analysed. The primary outcome was all-cause mortality. In ICD patients, the co-primary outcome of first appropriate shock was used. Mean follow-up time was 2.4 ± 1.1 years to death and 2.3 ± 1.2 years to the first appropriate shock. Pathological Q waves were associated with decreased mortality in ICD patients [hazard ratio (HR) 0.54, 95% confidence interval (CI) 0.35–0.84; *P* < 0.01] and patients with pathological Q waves had significantly more benefit from ICD (HR 0.44, 95% CI 0.21–0.93; *P* = 0.03). QTc interval increase taken as a continuous variable was associated with both mortality and appropriate shock incidence, but commonly used cut-off values, were not statistically significantly associated with either of the outcomes.

**Conclusion:**

Pathological Q waves were a strong ECG predictor of ICD benefit in primary prophylactic ICD patients. Excess mortality among Q wave patients seems to be due to arrhythmic death which can be prevented by ICD.

What’s new?

- Electrocardiogram variables are useful in evaluating implantable cardioverter-defibrillator (ICD) benefit in primary prevention.
- Q waves are associated with significantly lower mortality among ICD patients compared to control patients without Q waves.
- Patients with prior transmural infarction and left ventricular ejection fraction under 35% seem to benefit the most from primary prevention ICD therapy.

## Introduction

For almost two decades, implantable cardioverter-defibrillator (ICD) has been routinely used for primary prevention to reduce the risk for sudden cardiac death (SCD). The current guidelines are mainly based on the landmark studies MADIT-II, SCD-HeFT, and DEFINITE, published at the beginning of the millennium.^1–3^ Primary prophylactic ICD is recommended in patients with ischaemic or non-ischaemic cardiomyopathy (ICM) and left ventricular ejection fraction (LVEF) not exceeding 35%.^4^ In the EU, this has resulted in more than 100 000 ICD implantations annually.^5^

Nevertheless, both pharmaceutical and invasive treatment as well as device programming have evolved, and it has been shown that all-cause mortality and appropriate shock rates have decreased significantly since the guidelines conception.^6^ In addition, the competing risk of non-arrhythmic death may reduce the ICD benefit.^7^ In contrast, up to one in four patients experiences considerable side effects from ICD therapy (such as device infections or inappropriate shocks). As a consequence, benefit–risk ratio of ICD implantation is becoming less favourable.^8^ This has led to wide discussion of primary prophylactic ICD indications. The recent large randomized DANISH ICD trial did not show statistically significant overall survival benefit of ICD in non-selected patients with non-ICM.^9^ The need becomes obvious for better identification of patients who truly benefit from primary prophylactic ICD implantation.

Standard 12-lead electrocardiogram (ECG) is an easy and affordable tool to assess the risk of SCD. We have previously shown a combination of ECG variables to be a useful tool in identifying low-risk ICD patients in a retrospective ICD cohort.^10^ However, large prospective, controlled cohorts are missing. In this study, we assessed the prognostic value of ECG variables in the EU-CERT-ICD prospective cohort. We hypothesized that one or more variables derived from standard 12-lead ECG might help to assess the treatment benefit among primary prophylactic ICD patients.

## Methods

### Study design

EU-CERT-ICD (EUropean Comparative Effectiveness Research to assess the use of primary prophylacTic Implantable Cardioverter-Defibrillators) is a non-randomized, controlled, prospective multicentre study (ClinicalTrials.gov Identifier: NCT02064192). The study was investigator-initiated and was funded by the European Community’s Seventh Framework Programme (FP7). The study protocol and the prospective study objectives have been previously published in detail.^11^ The present analysis of ECG variables formed a Work Package 7 and was based on the original research plan within the EU-CERT-ICD framework.

The study was approved by local ethics committees at all participating centres. All patients provided written informed consent before inclusion. The study was conducted in accordance with the Declaration of Helsinki and Good Clinical Practice principles.

### Study population and outcomes

The prospective EU-CERT-ICD study cohort includes two treatment groups: ICD group of patients receiving primary prophylactic ICD implantation and a control group of patients receiving conservative treatment. All patients were candidates for primary prophylactic ICD treatment according to the current guidelines. Minimum age to be enrolled to the study was 18 years, LVEF was required to be ≤35%, and New York Heart Association functional class (NYHA class) was II or III (or NYHA Class I and LVEF ≤30%). Exclusion criteria were indication for secondary prophylactic ICD, indication for cardiac resynchronization therapy, pacemaker implanted, high-degree atrioventricular block (>II), unstable cardiac conditions such as NYHA Class IV or acute coronary syndrome, or life-expectancy of 1 year or less. The number of patients with atrial fibrillation (AF) was limited to 15%. The control group was required to fulfil the same inclusion and exclusion criteria and the control patients also received optimal conservative therapy.

In the ICD group, ICDs were implanted according to local practice at individual centres. Implantable cardioverter-defibrillator programming was consistent between participating centres, and included ventricular tachycardia therapy zone, ventricular fibrillation therapy zone, and a monitor zone. Ventricular tachycardia was treated by antitachycardia pacing (ATP) followed by shocks of maximum output. Ventricular fibrillation was treated by ATP during charge (if applicable) and shocks of maximum output. Implantable cardioverter-defibrillator programming could be individualized by the physician on clinical grounds.

Both patient groups were followed up regularly. Implantable cardioverter-defibrillator patients every 3–6 months and control patients every 6–12 months. Episodes of shock or ATP were stored as electrograms for adjudication, and programming changes were recorded.

Documented clinical variables included underlying cardiac disease, NYHA functional class, heart rate, resting blood pressure, weight, height, cardiovascular pharmacological treatment, peripheral arterial disease, cerebral vascular disease, pulmonary disease, diabetes mellitus, hypertension, sleep apnoea, tobacco use, any malignant disease, and standard laboratory parameters including creatinine, estimated glomerular filtration rate, serum blood urea nitrogen, and N-terminal prohormone of brain natriuretic peptide or BNP.

The primary outcome of the study reported here was all-cause mortality. The co-primary outcomes were time-to-first appropriate shock and ICD benefit. All-cause mortality and first appropriate shock were reviewed by the external committee which provided blind adjudication. Implantable cardioverter-defibrillator shocks were adjudicated after review of device electrograms and classified as appropriate or inappropriate. Minimum follow-up time 1 year was used in the present investigation.

### Electrocardiography assessment

A high-resolution 12-lead ECG was recorded at enrolment. Electrocardiogram data were digitally stored at the University of Göttingen (Göttingen, Germany). Data pre-processing was done at the Technical University of Munich. Analyses were performed blinded to outcomes at the University of Oulu (Oulu, Finland), using custom-made ECG analysis software (EASE, Oulu University Hospital and University of Oulu, Oulu, Finland).^12^

Electrocardiogram analysis included both traditional and novel ECG variables. Traditional ECG variables included QRS duration (QRSd), ventricular conduction delays, heart rate, heart rate-corrected QT intervals (QTc with Framingham’s formula), pathological Q waves, T-wave inversions, and rhythm (sinus rhythm, AF). Novel ECG variables included QRS-complex fragmentation (fQRS) and early repolarization (ER).^13^^,^^14^ QRS duration was analysed both as a continuous variable and as a categorical variable with a cut-off point 120 ms.^15^ In addition, QTc (corrected with Framingham’s formula) was analysed both as a continuous variable and as a categorical variable with a cut-off point 450 ms in male and 470 ms in female.^16^ Ventricular conduction delays were divided into left bundle branch block, right bundle branch block, and non-specific intraventricular conduction delay according to American Heart Association (AHA) recommendation.^17^ T-wave end was defined as the latest point where the T-wave reached the isoelectric line. Q waves were assessed manually according to the Minnesota code.^18^ Two or more large Q waves in two contiguous leads were cited as pathological. The presence of T-wave inversion was defined as T-wave negative by 0.1 mV or more in leads other than aVR, III, or V1. fQRS was analysed according to our own criteria^13^ in patients with QRSd ≤120 ms and according to Das *et al.*^19^ in patients with QRSd equal to 120 ms or more. Early repolarization was defined based on a recent consensus paper of MacFarlane *et al*.^14^ The territory of the Q waves, T-wave inversions, and fQRS was defined as anterior (V1–V3), inferior (II, III, aVF), or lateral (I, aVL, V4–V6), while in ER we excluded anterior leads V1–V3 from analyses to avoid confusion with the Brugada pattern.

### Statistical analysis

Continuous variables are presented as means and standard deviations, categorical variables as absolute and relative frequencies. Cox regression model was used to analyse the time-dependent probability of all-cause mortality. The time to first appropriate shock was analysed using a Fine and Gray competing risk model accounting for death, heart transplantation, and implantation of a ventricular assistant device as events competing to appropriate shocks. Results on survival models are reported using hazard ratio (HR) with 95% confidence intervals (CI). A *P*-value below 0.05 was considered statistically significant.

Predictors of mortality were assessed among both groups, while predictors of first appropriate shock were assessed in ICD treatment group only. The aim was to adjust the analyses of individual ECG markers for clinical prognostic factors. The latter were identified by a stepwise variable selection procedure with *P*-value <0.1 for entry and stay resulting in a multivariable model that we refer to as the ‘base model’. Individual ECG markers were then added one by one to the relevant base model. This means that the HRs of the ECG markers are adjusted for the variables of the base model but not for other ECG markers. All multiple analyses were adjusted and stratified by region (Eastern Europe: Hungary, Bulgaria, Croatia, Poland, Slovakia, and the Czech Republic; Western Europe: Germany, Belgium, Netherlands, and Switzerland; Northern Europe: Denmark, Sweden, Finland; and Southern Europe: Spain, Greece).

For the assessment of interactions between ECG variables of interest and ICD benefit, we first calculated a propensity score based on all baseline variables of interest. Two subsequent adjustments for the propensity score were used: stratification by propensity score quintiles (*n* = 2073) and a matched analysis of 1398 patients (2 ICD patients for each control patient, 2:1 matching) with similar baseline characteristics based on the propensity score. In a second step, Cox regression analyses of all-cause mortality were conducted including treatment (ICD vs. control), individual ECG variables of interest, and their interaction with treatment. Details on the derivation of the propensity score can be found in Bauer *et al.*^20^ including the Supplementary material online, *Appendix*.

## Results

### Baseline characteristics

**Figure 1:**
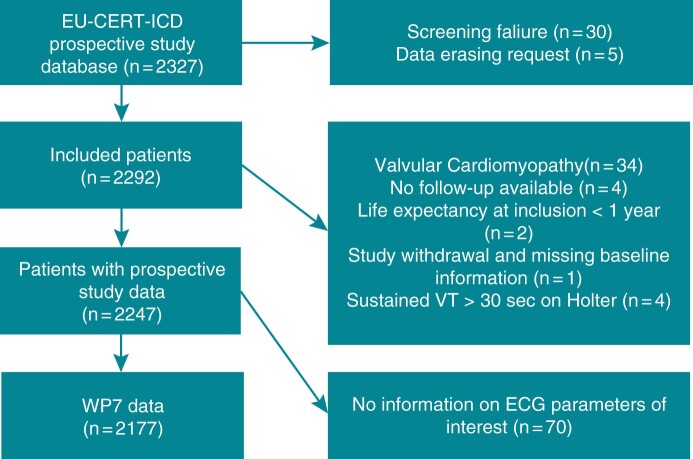
Flow chart of study data. ECG, electrocardiogram; VT, ventricular tachycardia; WP7, Work Package 7.

The participants were enrolled at 44 centres across 15 EU countries between 12 May 2014 and 7 September 2018. In total, 2327 patients with ICM or dilated cardiomyopathy were recruited; 1553 patients with ICD implantation and 774 control patients. The final population of our study included 2177 patients (18.2% female), 1477 patients (67.9%) in the ICD group and 700 patients (32.1%) in the control group; the flowchart is shown in *Figure 1*. At the time of the implantation, mean age was 62.4 ± 11.6 years. A total of 329 deaths (15.1%) occurred during mean follow-up time (2.4 ± 1.1 years) to death or censoring. A total of 105 patients (7.1%) received their first appropriate shock during mean follow-up time (2.3 ± 1.2 years) to appropriate shock, death, or censoring. Baseline characteristics are shown in *Table 1*.

**Table 1:** Baseline characteristics

	ICD group (1477), *N* (%)	Control group (700), *N* (%)	Total (2177), *N* (%)
Female	269 (18.2)	127 (18.1)	396 (18.2)
Age (years)	61.9 (11.5)	63.5 (11.7)	62.4 (11.6)
BMI (kg/m^2^)	27.8 (5.2)	28.2 (4.9)	27.9 (5.1)
Creatinine (mg/dL)	1.15 (0.58)	1.23 (0.61)	1.18 (0.59)
Diastolic blood pressure (mmHg)	74.0 (11.1)	75.2 (11.1)	74.4 (11.1)
Haemoglobin	13.8 (1.8)	13.8 (1.8)	13.8 (1.8)
Sodium (mmol/L)	139.1 (3.2)	139.4 (3.2)	139.2 (3.2)
LVEF (%)	27.5 (5.5)	29.1 (5.5)	28.0 (5.6)
QTc (ms)	441.6 (31.7)	443.7 (35.3)	442.3 (32.9)
QRS (ms)	111.8 (20.0)	114.1 (22.0)	112.5 (20.7)
Diabetes	443 (30.0)	215 (30.7)	658 (30.2)
COPD	170 (11.5)	68 (9.7)	238 (10.9)
Leading cardiac disease			
Ischaemic cardiomyopathy	1020 (69.1)	396 (56.6)	1416 (65.0)
Dilated cardiomyopathy	457 (30.9)	304 (43.4)	761 (35.0)
NYHA class			
Class I or II	927 (62.8)	394 (56.3)	1321 (60.7)
Class III or IV	550 (37.2)	306 (43.7)	856 (39.3)
Tobacco use	952 (64.5)	330 (47.1)	1282 (58.9)
Amiodarone	113 (7.7)	107 (15.3)	220 (10.1)
AT1 antagonist	282 (19.1)	176 (25.1)	458 (21.0)
Beta-blocker	1397 (94.6)	655 (93.6)	2052 (94.3)
Loop diuretic	1035 (70.1)	536 (76.6)	1571 (72.2)
FU until death or censoring	2.7 (1.0)	1.7 (1.2)	2.4 (1.1)
FU until first app. shock, death, or censoring	2.6 (1.0)	1.7 (1.2)	2.3 (1.2)
Death	218 (14.8)	111 (15.9)	329 (15.1)
First appropriate shock	105 (7.1)	–	–

The values are depicted as mean (SD) or counts (percentages).

BMI, body mass index; COPD, chronic obstructive pulmonary disease; FU, follow-up time (years); ICD, implantable cardioverter-defibrillator; LVEF, left ventricular ejection fraction; NYHA, New York Heart Association; QTc, QT-interval corrected by Framingham’s formula; SD, standard deviation.

*Table 2* shows baseline characteristics of analysed ECG variables. A total of 1542 patients (70.8%) had normal QRSd (QRSd < 120 ms) and 1155 patients (53.1%) had normal QTc (QTc <450 ms in male and <470 ms in female). A total of 1858 ECGs (85.3%) showed sinus rhythm. Pathological Q waves were identified in 407 patients (18.7%), T-wave inversion in 1369 patients (62.9%), ER in 229 patients (10.5%), and fQRS in 879 patients (40.4%) (*Table 2*).

**Table 2:** Baseline characteristics, categorical ECG variables

	ICD group (1477), *N* (%)	Control group (700), *N* (%)	Total (2177), *N* (%)
Ventricular conduction			
Normal ventricular conduction	1069 (72.4)	473 (67.6)	1542 (70.8)
LBBB	38 (2.6)	33 (4.7)	71 (3.3)
RBBB	116 (7.9)	62 (8.9)	178 (8.2)
IVCD	254 (17.2)	132 (18.9)	386 (17.7)
QRS < 120	1069 (72.4)	473 (67.6)	1542 (70.8)
QTc < 450 (male)/470 (female)	797 (54.0)	358 (51.1)	1155 (53.1)
Sinus rhythm	1286 (87.1)	572 (81.7)	1369 (62.9)
AF (history or present)	361 (24.4)	200 (28.6)	561 (25.8)
Q_inf	62 (4.2)	18 (2.6)	80 (3.7)
Q_lat	61 (4.1)	16 (2.3)	77 (3.5)
Q_ant	170 (11.5)	82 (11.7)	252 (11.6)
ER_inf	122 (8.3)	50 (7.1)	172 (7.9)
ER_lat	48 (3.2)	21 (3.0)	69 (3.2)
fQRS_ant	107 (7.2)	48 (6.9)	155 (7.1)
fQRS_lat	379 (25.7)	132 (18.9)	511 (23.5)
Tinv_ant	406 (27.5)	161 (23.0)	567 (26.0)
Tinv_inf	781 (52.9)	388 (55.4)	1169 (53.7)
Tinv_lat	332 (22.5)	172 (24.6)	504 (23.2)
Q_tot	291 (19.7)	116 (16.6)	407 (18.7)
Tinv_tot	918 (62.2)	451 (64.4)	1369 (62.9)
ER_tot	161 (10.9)	68 (9.7)	229 (10.5)
fQRS_tot	635 (43.0)	244 (34.9)	879 (40.4)

The values are depicted as counts (percentages).

AF, atrial fibrillation; ant, anterior; ECG, electrocardiogram; ER, early repolarization; fQRS, QRS-complex fragmentation; ICD, implantable cardioverter-defibrillator; inf, inferior; IVCD, non-specific intraventricular conduction delay; lat, lateral; LBBB, left bundle branch block; Q, pathological Q wave; QTc, QT-interval corrected by Framingham’s formula; RBBB, right bundle branch block; Tinv, T-wave inversion; tot, total.

There are some differences in baseline characteristics between ICD group and control group, but groups are mostly well matched and comparable (*Tables 1* and *2*). The differences between groups were considered in the statistical analysis.

### Predictors of outcomes

In ICD patients, AF, Q waves, anterior Q waves, and lateral fQRS showed statistically significant influences on all-cause mortality. Atrial fibrillation (HR 1.42, 95% CI 1.07–1.90; *P* = 0.02) and lateral fQRS (HR 1.50, 95% CI 1.12–2.00; *P* < 0.01) increased the risk for mortality, while pathological Q waves (HR 0.54, 95% CI 0.35–0.84; *P* < 0.01) and pathological anterior Q waves (HR 0.40, 95% CI 0.21–0.76; *P* < 0.01) were associated with a reduced risk of death among ICD patients (*Table 3*). In control patients, QTc and QRSd as continuous variables, as well as categorical variables QTc with cut-off point 450 ms in male and 470 ms in female and QRSd with cut-off point 120 ms were predictive of all-cause mortality. Prolonged QTc (HR 1.02, 95% CI 1.01–1.02; *P* < 0.01), as well as wide QRSd (HR 1.01, 95% CI 1.00–1.02; *P* = 0.03), QTc ≥450 ms in male and ≥470 ms in female (HR 1.77, 95% CI 1.15–2.74; *P* = 0.01) and QRSd ≥120 ms (HR 1.61, 95% CI 1.06–2.44; *P* = 0.03) increased the risk of death among control patients (*Table 4*).

**Table 3:** Multiple Cox regression model stratified by region on death in ICD patients including single variables of interest

	HR	95% CI	*P*-value
**Age (years)**	1.03	1.01–1.05	<0.01
**BMI (kg/m^2^)**	0.97	0.94–1.00	0.03
**Chronic obstructive pulmonary disease (yes vs. no)**	2.33	1.71–3.19	<0.01
eGFR	0.99	0.99–1.00	0.07
**Haemoglobin (g/dL)**	0.88	0.81–0.96	<0.01
Loop diuretic (yes vs. no)	1.37	0.95–1.99	0.09
**LVEF (%)**	0.95	0.93–0.97	<0.01
**NYHA (Class III or IV vs. Class I or II)**	1.65	1.25–2.20	<0.01
**PAD (yes vs. no)**	1.56	1.10–2.21	0.01
Sex (male vs. female)	1.43	0.98–2.10	0.06
**Sodium (mmol/L)**	0.93	0.90–0.97	<0.01
Adjusting for the variables shown above and stratifying by region, we observe the following results for each parameter individually			
QRSd (ms)	1.01	1.00–1.01	0.09
Heart rate (b.p.m.)	1.01	0.10–1.02	0.15
QTc	1.00	1.00–1.01	0.08
**Q_ant (yes vs. no)**	0.40	0.21–0.76	<0.01
**fQRS_lat (yes vs. no)**	1.50	1.12–2.00	<0.01
**Q_tot (yes vs. no)**	0.54	0.35–0.84	<0.01
**Atrial fibrillation (yes vs. no)**	1.42	1.07–1.90	0.02
QRS120 (yes vs. no)	1.30	0.98–1.73	0.07
IVCD (yes vs. no)	1.28	0.94–1.76	0.12
QTc 450/470 (yes vs. no)	1.20	0.89–1.63	0.23
Sinus rhythm (yes vs. no)	0.83	0.58–1.18	0.30

*n* = 1444, statistically significant results presented in bold.

95% CI, 95% confidence interval; BMI, body mass index; eGFR, estimated glomerular filtration rate; fQRS, QRS-complex fragmentation; HR, hazard ratio; ICD, implantable cardioverter-defibrillator; IVCD, non-specific intraventricular conduction delay; LVEF, left ventricular ejection fraction; NYHA, New York Heart Association; PAD, peripheral artery disease; Q, pathological Q wave; QRS120, QRS duration ≥120 ms (1) or less 0; QRSd, QRS duration; QTc 450/470, QTc ≥450 ms in male and ≥470 ms in female (1) or less 0; QTc, QT-interval corrected by Framingham’s formula.

**Table 4:** Multiple Cox regression model stratified by region on death in control patients including single variables of interest

	HR	95% CI	*P*-value
**Age (years)**	1.04	1.02–1.06	<0.01
**BMI (kg/m^2^)**	0.93	0.89–0.98	<0.01
**Creatinine (mg/dL)**	1.30	1.06–1.60	0.01
**Diastolic blood pressure (mmHg)**	0.98	0.96–1.00	0.02
Haemoglobin (g/dL)	0.90	0.79–1.02	0.09
Heart rate (b.p.m.)	1.01	1.00–1.03	0.06
**LVEF (%)**	0.92	0.89–0.95	<0.01
**Sex (male vs. female)**	2.27	1.25–4.12	<0.01
Sodium (mmol/L)	1.06	0.10–1.13	0.07
Adjusting for the variables shown above and stratifying by region, we observe the following results for each parameter individually			
**QTc**	1.02	1.01–1.02	<0.01
**QRSd (ms)**	1.01	1.00–1.02	0.03
**QTc 450/470 (yes vs. no)**	1.77	1.15–2.74	0.01
**QRS120 (yes vs. no)**	1.61	1.06–2.44	0.03
IVCD (yes vs. no)	1.47	0.94–2.30	0.09

*n* = 666, statistically significant results presented in bold.

95% CI, 95% confidence interval; BMI, body mass index; HR, hazard ratio; IVCD, non-specific intraventricular conduction delay; LVEF, left ventricular ejection fraction; QRS120, QRS duration ≥120 ms (1) or less 0; QRSd, QRS duration; QTc 450/470, QTc ≥450 ms in male and ≥470 ms in female (1) or less 0; QTc, QT-interval corrected by Framingham’s formula.

Multiple competing risk model showed that only QTc as a continuous variable demonstrated a significant effect on first appropriate shock in a multiple analysis accounting for baseline covariates. Prolonged QTc increased the probability for the first appropriate shock (HR 1.01, 95% CI 1.00–1.01; *P* = 0.02). Nevertheless, abnormal QTc intervals defined above (the cut-off 450 ms for males and 470 ms for females) were not associated with increased risk of ICD shocks (*Table 5*).

**Table 5:** Multiple Fine and Gray competing risk model on first appropriate in ICD patients stratified by region including ECG parameters of interest (*n* = 1465)

	HR	95% CI	*P*-value
**Digitalis glycoside (yes vs. no)**	2.56	1.50–4.37	<0.01
**BMI (kg/m^2^)**	1.04	1.01–1.08	<0.01
**Sex (male vs. female)**	2.47	1.25–4.88	<0.01
**Chronic obstructive pulmonary disease (yes vs. no)**	1.75	1.06–2.90	0.03
Including single parameters into the model above leads to the following results			
**QTc (ms)**	1.01	1.00–1.01	0.02
Atrial fibrillation (yes vs. no)	1.50	0.97–2.31	0.07
Sinus rhythm (yes vs. no)	0.67	0.39–1.17	0.16

*n* = 1465, statistically significant results presented in bold.

95% CI, 95% confidence interval; BMI, body mass index; ECG, electrocardiogram; HR, hazard ratio; ICD, implantable cardioverter-defibrillator; QTc, QT-interval corrected by Framingham’s formula.

### Predictors of implantable cardioverter-defibrillator benefit

*Table 6* shows interactions of ECG parameters with the ICD effect in propensity score-adjusted Cox regressions for all-cause mortality as endpoint. QTc as continuous variable, Q wave in any location, and anterior Q wave were associated with benefit from ICD treatment. Patients with prolonged QTc appeared to benefit from ICD, but QTc prolongation evaluated according to the QTc cut-offs was not associated with ICD benefit. Patients with any Q wave (HR 0.44, 95% CI 0.21–0.93; *P* = 0.03) or with anterior Q wave (HR 0.37, 95% CI 0.14–0.96; *P* = 0.04) did significantly benefit from ICD more than patients without Q waves (*Table 6*).

**Table 6:** Interactions of ECG parameters with ICD effect in propensity score-adjusted Cox regressions for all-cause mortality

	Stratified by propensity score quintiles (*n* = 2073)			2:1 matching (*n* = 1398)		
	HR	95% CI	*P*-value	HR	95% CI	*P*-value
**ICD effect (ICD vs. control)**	0.69	0.53–0.90	<0.01	0.70	0.54–0.92	0.01
Interaction with ICD effect	HR interaction	95% CI	*P*-value	HR interaction	95% CI	*P*-value
**QTc**	0.99	0.98–1.00	<0.01	0.99	0.90–1.00	0.02
Heart rate (b.p.m.)	1.01	1.00–1.03	0.09	1.01	1.00–1.03	0.16
QRSd (ms)	1.00	0.99–1.01	0.52	1.00	0.99–1.01	0.70
ER_lat (yes vs. no)^a^	–	–	–	–	–	–
**Q_tot (yes vs. no)**	0.35	0.18–0.69	<0.01	0.44	0.21–0.93	0.03
**Q_ant (yes vs. no)**	0.29	0.13–0.69	0.01	0.37	0.14–0.96	0.04
Tinv_lat (yes vs. no)	0.61	0.35–1.08	0.09	0.92	0.48–1.73	0.79
Q_lat (yes vs. no)	0.33	0.08–1.44	0.14	0.39	0.08–2.00	0.26
fQRS_tot (yes vs. no)	1.32	0.80–2.17	0.27	1.35	0.77–2.35	0.30
QRS120 (yes vs. no)	0.79	0.49–1.28	0.34	0.81	0.47–1.39	0.44
QTc 450/470 (yes vs. no)	0.79	0.49–1.29	0.35	0.91	0.53–1.57	0.74
IVCD (yes vs. no)	0.79	0.47–1.35	0.40	0.85	0.47–1.54	0.60
fQRS_ant (yes vs. no)	0.69	0.29–1.65	0.40	1.44	0.52–3.99	0.48
ER_tot (yes vs. no)	0.70	0.30–1.64	0.42	0.69	0.27–1.80	0.45
Sinus rhythm (yes vs. no)	0.80	0.44–1.45	0.46	1.14	0.59–2.23	0.70
fQRS_lat (yes vs. no)	1.23	0.70–2.16	0.47	0.88	0.47–1.66	0.70
ER_inf (yes vs. no)	0.74	0.31–1.79	0.51	0.68	0.47–1.55	0.45
Tinv_inf (yes vs. no)	0.87	0.51–1.51	0.63	0.86	0.47–1.55	0.61
Q_inf (yes vs. no)	1.68	0.20–13.81	0.63	2.04	0.23–17.73	0.52
Tinv_tot (yes vs. no)	0.88	0.53–1.46	0.63	1.07	0.61–1.88	0.82
Tinv_ant (yes vs. no)	0.92	0.56–1.49	0.72	0.99	0.57–1.69	0.96
LBBB (yes vs. no)	0.88	0.26–2.97	0.84	0.75	0.20–2.88	0.68
RBBB (yes vs. no)	0.94	0.44–2.04	0.88	0.93	0.38–2.25	0.87

Statistically significant results presented in bold.

95% CI, 95% confidence interval; ant, anterior; ECG, electrocardiogram; ER, early repolarization; fQRS, QRS-complex fragmentation; HR, hazard ratio; ICD, implantable cardioverter-defibrillator; inf, inferior; IVCD, non-specific intraventricular conduction delay; lat, lateral; LBBB, left bundle brunch block; Q, pathological Q wave; QRS120, QRS duration ≥120 ms (1) or less 0; QRSd, QRS duration; QTc 450/470, QTc ≥450 ms in male and ≥470 ms in female (1) or less 0; QTc, QT-interval corrected by Framingham’s formula; RBBB, right bundle branch block; Tinv, T-wave inversion; tot, total.

a All ICD patients with lateral ER (*N* = 69) survived the follow-up time.

**Figure 2:**
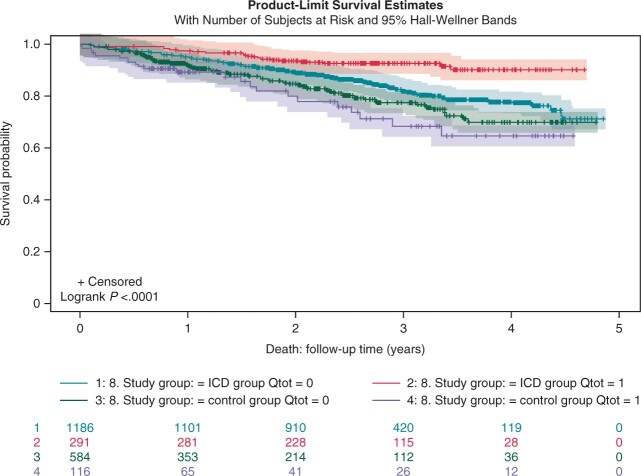
Mortality among subjects with and without Q waves in ICD and control populations. ICD, implantable cardioverter-defibrillator.

Benefit from ICD therapy regarding Q-wave status in both ICD and control populations are presented in *Figure 2*.

## Discussion

In this study, we analysed several traditional and novel variables derived from standard 12-lead ECGs in a prospective non-randomized study among EU-CERT-ICD patients. Our findings suggest that some of the ECG variables might be valuable in the evaluation of risk for all-cause mortality, as well as in the estimation of ICD benefit in primary prophylactic ICD patients.

Continuous variable of QTc duration was associated with ICD shocks in ICD patients and with death in control patients. However, QTc prolongation defined as a categorical variable with a cut-off point 450 ms in male and 470 ms in female did not perform this well. These results are in line with prior studies, although no fully comparable studies among primary prophylactic ICD patients are available. In general, QTc interval prolongation is a known risk factor for arrhythmic events and SCD.^21^

It has been previously shown that prolonged QRS (QRSd ≥120 ms) might predict sudden death and life-threatening arrhythmias in ICD patients.^22^ In our prospective data, QRS duration predicted death in control patients both when used as a continuous variable and when QTS prolongation was defined with a cut-off point of QRSd ≥120 ms. QRSd was not significant predictor of other outcomes. However, our findings support prior evidence that prolonged QRS is a risk marker among patients eligible for ICD treatment. In addition, while widened QRSd did not predict death in ICD patients, it may be assumed that ICD patients with QRSd have benefit from the device, despite the statistically non-significant results.

Several prior studies have reported that AF predicts appropriate shocks and death in primary prophylactic ICD populations.^23^ In the present study, AF was significantly prognostic only for all-cause mortality in ICD patients. It did not reach statistical significance in terms of appropriate shocks or ICD benefit.

Lateral fQRS was found to predict all-cause mortality in ICD patients in the present study. Prior studies on fQRS and prognosis of primary prophylactic ICD patients are conflicting. In the MADIT-II cohort, inferior fQRS predicted SCD and ICD shocks.^24^ In contrast, other studies have not found any association between fQRS and appropriate shocks or death.^25^ Hence, despite the observations in our study, the specific risk prediction role of fQRS remains unclear. Some of the conflicting results might have resulted from the lack of consensus fQRS definition. In this study, we used our own fQRS definition^13^ in patients with QRSd ≤120 ms and definition by Das *et al.*^19^ in patients with wide QRS.

The most important result in this study is that the presentation of pathological Q wave in any location and especially in anterior leads was protective of death in our ICD patient cohort. In addition, patients without any Q waves or without anterior Q wave did not benefit from ICD treatment. This is a novel finding of preferable prognosis associated with pathological Q waves. Q waves are known markers of an old myocardial infarction and are traditionally considered as a marker of poor prognosis.^26^ In previous landmark trials, such as MADIT-II and SCD-Heft, ECG markers of myocardial scarring were evaluated by means of Selvester score from 12-lead ECGs. In SCD-Heft, there was an association of Selvester score and prognosis, but corresponding results were not seen in MADIT-II.^27^^,^^28^ Using only Q-wave presence in our study was a deliberate decision, since we aimed at focusing on ECG variables that could easily be evaluated in clinical practice. To some extent, Q waves do not only illustrate that there is a scar in the myocardium, but also the reason and mechanism of heart failure which lead to implantation of primary prophylactic ICD. Ischaemic cardiomyopathy patients were shown to benefit more from ICD than non-ischaemic patients.^12^ The existence of Q waves is a clear marker of ischaemic aetiology, but the results were significant even after adjustment for ischaemic aetiology. As can be seen from *Figure*2, *Q*-wave patients benefit from ICD more than patients without Q waves, which possibly is a result of highest SCD risk among Q-wave patients and somewhat lower competing non-SCD mortality among these patients.

Based on our study, no single ECG marker measured from a standard 12-lead ECG should be used to make the decision on whether to implant an ICD or not. However, ECG variables are easy, inexpensive, and routinely recorded. They seem to provide additional information on ICD benefit and might thus be used for detailed personalized patient selection for ICD therapy. We have previously shown that combination of ECG variables might identify low-risk patients in real-life primary prophylactic ICD cohort.^10^ Combining several ECG variables or ECG variables with other variables, such as 24-h ECG recording or magnetic resonance imaging, might enhance the assessment for ICD treatment indications.

The main limitation of our study is the non-randomized study design. The decision to use strictly controlled, non-randomized study instead of randomized trial was based on ethical aspects considered during the EU-CERT-ICD design. However, ICD group and control group are very comparable in terms of baseline characteristics, and statistical methods compensated for remaining baseline differences. We therefore believe that the results of our study are valid. The follow-up time used in the study was rather short, particularly in control group. The results should be confirmed when longer follow-up time becomes available.

## Conclusion

Pathological Q waves were a strong ECG predictor of lower all-cause mortality among ICD patients. There is a major beneficial effect on ICD therapy among patients with Q waves compared to patients without Q waves probably due to the increased risk for arrhythmic death rather than competing risk for non-SCD among these patients.

## Supplementary material

Supplementary material is available at *Europace* online.

## Funding

The EU-CERT-ICD project is funded by the European Community’s 7th Framework Programme FP7/2007–2013 (602299). 

**Conflict** **of interest:** none declared.

## Data availability

The data underlying this article will be shared on reasonable request to the corresponding author.

## Supplementary Material

euab260_Supplementary_Data
