# Supplementary material for: Q waves are the strongest electrocardiographic variable associated with primary prophylactic implantable cardioverter-defibrillator benefit: a prospective multicentre study
Source: Europace. 2021 Nov 29;24(5):774–83. doi: 10.1093/europace/euab260 (PMC9071070; doi:10.1093/europace/euab260)
Supplement: euab260_Supplementary_Data [file euab260_supplementary_data.docx]

***List of EU-CERT-ICD centers and investigators***

Investigators - EU-CERT-ICD centres (grant beneficiaries) – usually two each

1, Elena Arbelo, MD

2,3,4,9 Axel Bauer MD

5, Frieder Braunschweig MD PhD

1, Josep Brugada MD, PhD

6, David Conen MD

7, Iwona Cygankiewicz MD

8, Michael Dommasch MD

9, Christian Eick, MD

10, Panagiota Flevari MD

11,12 Tim Friede PhD

13, Jan Galuszka MD

14, Jim Hansen MD

15, Robert Hatala MD

11, Markus Harden PhD

16, Katerina Hnatkova PhD

17, Heikki V. Huikuri MD

17, Juhani M. Junttila, MD PhD

2,3 Stefan Kääb MD

18, Gabriela Kaliska MD

19, Jaroslaw D. Kasprzak MD

10, Andreas Katsimardos MD

20, Milan Kozak MD

21, Tomasz Kuczejko MD

21, Andrzej Lubinski MD

18, Jozef Martinek PhD

22, Béla Merkely MD, PhD

20, Tomáš Novotný MD

16, Marek Malik PhD MD

22, Peter Perge MD

23, Burkert Pieske MD

24, Pyotr Platonov MD PhD

7, Pawel Ptaczyński, MD

19, Dariusz Qavoq MD

25, L. Rotkvić, MD

22, Zoltan Sallo MD

12,26, Simon Schlögl MD

4,7, Georg Schmidt MD

2,3 Moritz Sinner, MD

26, Rajeeva Sritharan MSc

27, Stefan Stefanow MD

28, Christian Sticherling MD PhD

29, Jesper Hastrup Svendsen MD DMSc

15, Martin Svetlosak MD

25, Janko Szavits-Nossan MD

13, Milos Taborsky MD

30, Anton Tuinenburg, MD

31, Bert Vandenberk MD

30, Marc A. Vos PhD

31, Rik Willems MD PhD

23, Stefan N Willich MD PhD

27, Christian Wolpert MD

12,26, Markus Zabel MD

Investigators - Third party clinical centers (usually one each)

32, Ante Anic, MD

32, Zoran Bakotic MD

33, Steffen Behrens MD

34, Dieter Bimmel MD

35, Sandro Brusich MD

36, Rüdiger Dissmann MD

37, Gerian Grönefeld, MD

38, Przemyzlav Guzik MD

39, Svetoslav Iovev MD

40, Zrinka Jurisic MD

34, Thomas Klingenheben, MD

41, Nikola Pavlović MD

42, Joachim Seegers MD

43, Robert H.G. Schwinger MD

44, Tchavdar Shalganov MD

45, Vassil Traykov MD

46, Vasil Velchev MD

EU-CERT-ICD centres/affiliations (grant beneficiaries)

1-IDIBAPS, Dept. of Cardiology, Hospital Clinic Barcelona, Spain

2-Dept. of Cardiology, Klinikum Großhadern, Ludwig-Maximilians-Universität Munich, Germany

3-German Center for Cardiovascular Research partner site Munich Heart Alliance, Munich, Germany

5-Dept. of Cardiology, Karolinska Institutet, Stockholm, Sweden

7-Dept. of Cardiology, Medical University of Lodz (MUL) CKD Hospital, Lodz, Poland

8-Med. Klinik und Poliklinik I, Technische Universität München, Klinikum rechts der Isar, Munich, Germany

9-Dept. of Cardiology, University Hospital Tübingen, Germany

10-2^nd^ Dept. of Cardiology, Attikon University Hospital, Athens, Greece

11-Dept. of Medical Statistics, University Medical Center Göttingen, Göttingen, Germany

12-DZHK (German Center for Cardiovascular Research), partner site Göttingen, Göttingen, Germany

13-Dept. of Cardiology, University Hospital, Olomouc, Czech Republic

14-Gentofte Hospital, Copenhagen, Denmark

15-Slovak Medical University NUSCH, Bratislava, Slovakia

16-National Heart and Lung Institute, Imperial College, London, United Kingdom

17-Medical Research Center, Oulu University Hospital and University of Oulu, Finland

18-Dept. of Cardiology, SUSSCH Banska Bystrica, Slovakia

19-Chair and Dept. of Cardiology, Bieganski Hospital, Medical University of Lodz (MUL), Lodz, Poland

20-Dept. of Internal Medicine and Cardiology, University Hospital Brno, Brno, Czech Republic

21-Dept. of Cardiology, Medical University of Lodz (MUL) WAM Hospital, Lodz, Poland

22-Dept. of Cardiology, Semmelweis University Heart Center, Budapest/Hungary

23-Institute for Social Medicine, Epidemiology and Health Economics and Dept. of Cardiology, Charité Universitätsmedizin Berlin, Berlin, Germany

24-Dept. of Cardiology, Lund University Hospital, Lund, Sweden

25-Dept. of Cardiology, Magdalena Klinika, Krapinske Toplice, Croatia

26-Dept. of Cardiology and Pneumology, Heart Center, University Medical Center, Göttingen, Germany

27-Dept. of Cardiology, Klinikum Ludwigsburg, Germany

28-Dept. of Cardiology, University Hospital, Basel, Switzerland

29-Dept. of Cardiology, The Heart Centre, Rigshospitalet, Copenhagen University Hospital, and Department of Clinical Medicine, University of Copenhagen, Copenhagen, Denmark

30-Dept. of Medical Physiology and Dept. of Cardiology, University Medical Center Utrecht

31-University Hospitals of Leuven, Leuven, Belgium

Third party EU-CERT-ICD clinical centers

32-Dept. of Cardiology, General Hospital, Zadar, Croatia

33-Dept. of Cardiology, Vivantes Humboldt Klinikum, Berlin, Germany

34-Dept. of Cardiology, Marienhospital, Bonn, Germany

35-Dept. of Cardiovascular disease, KBC Rijeka, Rijeka, Croatia

36-Dept. of Cardiology, Klinikum Reinkenheide, Bremerhaven, Germany

37-Dept. of Cardiology, Asklepios Klinikum Barmbek, Hamburg, Germany

38-Dept. of Cardiology, Poznan Medical University HSUH Hospital

39-Dept. of Cardiology, St. Ekaterina University Hospital, Sofia, Bulgaria

40-Dept. of Cardiology, KBC Split, Croatia

41-Dept. of Cardiology, KBC Sestre Milosrdnice, Zagreb, Croatia

42-Dept. of Cardiology, University Hospital Regensburg, Germany

43-Dept. of Cardiology, Klinikum Weiden, Germany

44-Dept. of Cardiology, National Heart Hospital, Sofia, Bulgaria

45-Dept. of Cardiology, Acibadem City Clinic Tokuda Hospital

46-Dept. of Cardiology, St. Anna Hospital, Sofia, Bulgaria

Further affiliations of EU-CERT-ICD investigators

4-University Hospital for Internal Medicine III, Medical University Innsbruck, Innsbruck, Austria

6-Population Health Research Institute, McMaster University, Hamilton, Ontario, Canada
